# Psychophysiological evaluation of the Smartick method in children with reading and mathematical difficulties

**DOI:** 10.3389/fnhum.2024.1287544

**Published:** 2024-04-04

**Authors:** César E. Corona-González, Moramay Ramos-Flores, Luz María Alonso-Valerdi, David I. Ibarra-Zarate, Victor Issa-Garcia

**Affiliations:** ^1^Tecnologico de Monterrey, Escuela de Ingeniería y Ciencias, Monterrey, Mexico; ^2^Facultad de Psicología, Benemérita Universidad Autónoma de Puebla, Puebla, Mexico

**Keywords:** learning difficulties, EEG, assistive technology, low academic performance, psychophysiological evaluation, Smartick

## Abstract

**Introduction:**

Assistive technologies for learning are aimed at promoting academic skills, such as reading and mathematics. These technologies mainly embrace mobile and web apps addressed to children with learning difficulties. Nevertheless, most applications lack pedagogical foundation. Additionally, the task of selecting suitable technology for educational purposes becomes challenging. Hence, this protocol posits the psychophysiological assessment of an online method for learning (OML) named Smartick. This platform comprises reading and math activities for learning training. In this protocol, individual monitoring of each child is proposed to determine the progress in learning caused by Smartick.

**Methods and analysis:**

One hundred and twelve children aged between 8 and 12 who present reading or math difficulty after a rigorous psychometric evaluation will be recruited. The study comprises four sessions. In sessions 1 and 2, collective and individual psychometric evaluations will be performed, respectively. Reading and mathematical proficiency will be assessed, as well as attentional levels and intellectual quotient. Subsequently, each child will be semi-randomly assigned to either the experimental or control groups. Afterward, a first EEG will be collected for all children in session 3. Then, experimental groups will use Smartick for 3 months, in addition to their traditional learning method. In contrast, control groups will only continue with their traditional learning method. Finally, session 4 will consist of performing a second psychometric evaluation and another EEG, so that psychophysiological parameters can be encountered that indicate learning improvements due to the OML, regardless of the traditional learning method at hand.

**Discussion:**

Currently, few studies have validated learning improvement due to assistive technologies for learning. However, this proposal presents a psychophysiological evaluation addressed to children with reading or math difficulties who will be trained with an OML.

## 1 Introduction

### 1.1 Assistive technology for learning

Elementary education allows children to develop basic cognitive skills for life, such as reading, writing, and calculation. For this reason, a wide variety of technologies have been designed to assist in the development or boosting of these abilities. Nevertheless, learning technologies often lack pedagogical foundation in their design (Ok et al., 2016; Papadakis, 2021). Unfortunately, there are no studies that validate the effects of these tools. Measuring the learning improvement is usually based on subjective parameters, such as opinion questionnaires or user experience. Conversely, there is a proliferation of digital applications on the internet (Papadakis and Kalogiannakis, 2017), and most of the download sites lack specificity at the description of these apps (Taylor et al., 2022). Therefore, the selection of an appropriate technology for learning is a challenging task for teachers, parents, or tutors. Lim and Toh (2022) proposed eight considerations to select an appropriate technology for learning:

• Determine what skills are to be developed (academic, social, or emotional)
• Learning methods, that can be oral/written, by positive feedback (giving rewards for succeeding activities), or collaborative, through co-teaching with parents or guardians
• Values encouraged by the interface
• Active interactivity with the technology for learning is highly recommended, instead of passive involvement (just watching).
• Gamification to increase motivation
• User friendly interface
• Age correspondence with the app
• Aesthetic interface design

Additionally, Noorhidawati et al. (2015) established that attention is necessary for a good performance within any app. Consequently, the learning app must fulfill three key points: (a) sensory stimulation (tactile, visual, auditory), (b) emotional expression, and (c) verbal expression. Once the user is engaged with the technology, learning comes from three directions:

• Cognitive direction, which encompasses mental processes, such as observation, recognition, comprehension and performing tasks within the app.
• Psychomotor direction, related with physical interaction with the system. Children must answer according to an incoming stimulus, either finger movement to select an appropriate option or the repetitiveness of actions that improve their performance.
• Affective direction associated with the emotional processing while using the app.

Currently, children show a growing interest in technology. By incorporating technology into the learning process, the ability to capture the child’s attention becomes faster, thereby enhancing the effectiveness of learning. In addition, there are advantages and disadvantages (Allison Academy, 1983; Brown, 2019) that must be considered about these technologies:

• Advantages

○ Attracts attention
○ Offers different learning strategies
○ Learning motivation
○ Each child learns at their own pace
○ Learning difficulties are shown easily

• Disadvantages

○ Tends to distract students
○ Social interaction is diminished
○ It can be used just for entertainment purposes
○ Excessive use of technology
○ Sustaining a learning technology can be expensive

Nowadays, some assistive technologies (AT) have been developed around the world to improve learning cognitive skills in children. These technologies embrace different mobile and web apps, augmented reality (AR) interfaces and virtual reality (VR) applications. AT for learning allows children to relieve anxiety while learning occurs in a playful way (Bryant et al., 2014). Additionally, some studies have found that using AT for learning can increase independent learning (Lindeblad et al., 2017), and enhance life functioning (Courtad and Bouck, 2013). Table 1 shows some works related to AT for learning.

**TABLE 1:** State of the art: assistive technology for learning.

Learning difficulty	Name		Country of design	Studied elements
Reading	Dyslexia Baca (Daud and Abas, 2013)		Malaysia	• Letter differentiation: “p,” “q,” “b,” “d,” “m,” and “w”
	DytectiveU (Rello et al., 2017)		Spain	• Alphabetic and phonological awareness • Spelling • Semantics
	EasyLexia 2.0 (Skiada et al., 2014)		Greece	• Orthography, phonology, and vocabulary • Image-word matching • Recalling numbers, letters and images
	Namagi (Schulte-Körne et al., 2016)		Germany	• Phonetic perception • Grapheme-phoneme correspondence • Reading literacy
	Alphabetics (Medcalf Laura, 2014)		USA	• Phonetic awareness • Shape recognition
	AlphaTots (Little 10 Robots, 2018)		USA	• Alphabet
	GraphoGame (University of Jyväskylä, 1993)		Finland	• Early literacy skills
	AR	NS (Tenemaza et al., 2019)	Ecuador	• Sound identification of the beginning of pseudowords • Reversion of words
		NS (Gupta et al., 2019)	India	• Real time text detection • Font style customization • Bigger text options • Out loud reading by the mobile app
		Interactive text book (Vinumol et al., 2013)	India	• Visual representation of words
		NS (Ho et al., 2011)	Hong Kong	• Learning vocabulary
	VR	NS (Maskati et al., 2021)	Saudi Arabia	• Tracing and pronouncing the Arabic alphabet
		NS (Rodríguez-Cano et al., 2021)	Spain	• Grapheme-phoneme correspondence • Syllable awareness • Lexical awareness • Association between numbers and images • Sound identification of consonants and words • Lexical development
Reading and math	Cognifit (Breznitz, 2024)		NS	• Training programs to enhance brain plasticity
	Starfall (Schutz, 2002)		USA	• Phonetics • Calculation
Math	Talasia (Kuhn and Schwenk, 2011)		Germany	• Magnitude comparison • Number words and digits • Numeric representation (read and written) • Cardinality • Addition and subtraction
	Calculic kids (Ariffin et al., 2019)		Malaysia	• Number and object counting • Addition and subtraction
	MathFun (Rohizan et al., 2020)		Malaysia	• Increase curiosity for learning math
	Coolmath4kids (Coolmath.com Llc, 2017)		USA	• Arithmetic • Fractions
	Calcularis (Käser et al., 2013)		Switzerland	• Spatial number representation • Arithmetic • Motivation for math • Knowledge for numbers up to 1,000
	AR	NS (Kellems et al., 2020)	USA	• Addition and subtraction of integers • Multiplication and division of integers • Ratios for basic unit conversion • Multiplication and division for rate changes
		NS (Miundy et al., 2020)	Malaysia	• Mathematical learning ability • Mathematical learning performance
		NS (Avila-Pesantez et al., 2019)	Ecuador	• Matching numbers
	VR	NS (Hwang and Hu, 2013)	Taiwan	• Surface and volume calculation

Various digital apps have been designed in different countries to assist children who suffer from learning difficulties. NS, not specified; AR, augmented reality; VR, virtual reality.

Finally, the Smartick^1^ company developed an online method for learning for the improvement of reading and mathematical skills in children. This technology is intended for both typically developed children (TDC) and those with special educational needs. Smartick has two interfaces (reading and mathematics), which are based on artificial intelligence (AI) that allows adjusting difficulty of activities according to each child capability (Arroyo and González de Vega, 2009).

### 1.2 EEG patterns in reading and mathematical process

Electroencephalography (EEG) has been utilized in several studies to compare brain activity in individuals with and without reading or mathematical difficulties. EEG spectral analysis is a technique that has been widely used to identify neural correlates in the processing of reading and mathematics. Pavithran et al. (2019) suggested that an increment in theta/alpha ratio in dyslexics children is related with inability to sustain oriented attention in reading exercises. Additionally, Jäncke and Alahmadi (2016) found an increment in theta/alpha ratios across central and posterior regions, along with augmented theta/beta values throughout frontal and central recording sites in learning-disabled children. Also, higher theta/alpha and theta/beta ratios were elicited from resting-state EEG in children with attention deficit hyperactivity disorder with concomitant reading disabilities (Clarke et al., 2002). In the same line, ratio of theta/alpha band power has been utilized to investigate mathematical difficulties (Nassehi et al., 2020). Similarly, it has been demonstrated that frontal alpha asymmetry and frontal theta are well-known neural markers regarding the complexity of solving mathematical operations and problem solving, respectively (Formaz, 2023).

On the other hand, different brain regions have been found to be involved in reading and math processing. Arns et al. (2011) identified increments in delta and theta bands on frontal and right temporal regions, when comparing resting state EEG in dyslexic and healthy infants. These findings agree with Nora et al. (2021), who suggested that there are right hemispheric pathways that could compensate neural impairments in the processing of reading, despite the extensive literature supporting the role of the left hemisphere in language processing. However, higher alpha and beta power have been identified in dyslexic children over centroparietal cortex, during a writing task (Che Wan Fadzal et al., 2012a,b). As noted by Soltanlou et al. (2019), an increase in theta band in frontal and parietotemporal regions are associated with arithmetic problem solving and mathematical information retrieval, respectively. Other techniques have also been applied to differentiate EEG patterns between neurotypical children from those with learning difficulties. This information is shown in Table 2.

**TABLE 2:** Previous studies: EEG patterns in learning difficulties.

Method	Study description	Result	References
Approximate entropy	Comparing dyslexic and controls.	No clear patterns were identified to differentiate between dyslexic and TDC.	Andreadis et al., 2009
SVM and ERP	Classification between dyslexic children and skilled reader by extracting temporal, spectral, and statistical features.	The left frontal region exhibited the greater differences between groups.	Frid and Manevitz, 2018
SVM	Contrasting writing and typing performance between dyslexic and non-dyslexic adults by the calculation of statistical features in each EEG band.	Prefrontal and frontal electrodes successfully differentiate between writing and typing groups, respectively, from the control group.	Perera et al., 2018
Coherence	Estimating differences in brain functional connectivity between children with opposite math proficiency (high and low).	Higher coherence on the right hemisphere for both groups. Moreover, high performers elicited higher local beta activity over parietal areas, whereas low performers showed a more extended activity toward frontoparietal region involving delta, theta, alpha and beta bands.	González-Garrido et al., 2018
SVM and wavelet	Estimation of theta and beta power ratio by using Daubechies, Symlets and Coiflets families. Then, a SVM classifier was utilized to contrast between normal, poor, and capable dyslexic children in a reading and writing task.	Classification accuracy of 91% when using theta and beta power ratio as an input feature to differentiate between normal, poor, and capable dyslexic children.	Zainuddin et al., 2018
		Area Under the Curve as a measure of classifier performance between normal, poor, and capable dyslexic children.	Mahmoodin et al., 2019
ERP	Poor and good arithmetical performers solved operations that showed two results: (a) correct/incorrect numerical value and a (b) congruent/incongruent finger representation. Participants were asked to answer according to the numerical value. The arithmetical performance was estimated by the P300 component.	Higher amplitudes in P300 when detecting correct answers, regardless of congruent/incongruent finger representation.	Proverbio and Carminati, 2019
	Assessing of arithmetical retrieval through P300 component among high, average, and low performers.	The higher the arithmetical performance was, the higher the P300 amplitude.	Gómez-Velázquez et al., 2022
	Searching for differences in N400 component between TDC and children with reading difficulties.	TDC exhibited higher amplitudes in N400 component than non-TDC, when words and nonwords were processed.	Sun et al., 2023

Different analyses have been applied to differentiate neural activity between individuals with and without learning difficulties. TDC, typically developed children; SVM, support vector machine; ERP, event related potentials.

### 1.3 Protocol purposes

This protocol formally posits the proposal for a comprehensive psychophysiological assessment of an online method for learning (OML) referred to as Smartick (Arroyo and González de Vega, 2009). Smartick is an AI-technology that adapts in real-time to the needs of each child, which allows learning improvements in accordance with the child’s capabilities. The assessment will comprise: (a) the application of psychometric tests that evaluate reading and mathematical abilities, as well as attentional levels and intellectual quotient (IQ), and (b) EEG signal analysis before and after the use of Smartick. The project is focused on children between third and sixth grade of elementary school with low academic performance, either in reading or mathematics.

## 2 Methods and analysis

### 2.1 Sample

One hundred and twelve children from third to sixth grade will be recruited, based on the following selection criteria depicted in Table 3. Sample size was estimated using G*Power^2^ software (v3.1.9.7) for a two-sample independent *t*-test (effect size *d* = 0.8, and confidence level 95%). The participants must show low academic performance in reading or mathematics. If both learning difficulties are exhibited, only the learning area with the greatest academic lag will be considered. These difficulties will be detected in two stages. First, three psychometric tests will be performed collectively: (a) d2-R test, to assess attention capacity (Brickenkamp et al., 2022), (b) PREDISCAL, to evaluate reading comprehension and mathematics (Pina et al., 2020), and (c) a dictation task, for orthography assessment (Zhang et al., 2020). Then, each child will be evaluated individually, according to the learning difficulty identified in the previous stage. For reading evaluation, three characteristics will be considered: (a) reading speed, (b) errors in reading, and (c) reading comprehension. The reading proficiency will be determined according to the manual of procedures for the promotion and evaluation of reading competence in the classroom (MPPERCC). This document was published in 2012 by the Secretariat of Public Education in Mexico, and it states that these three key factors summarize the reading performance in children from first grade of primary to third grade of secondary school (Secretaría de Educación Pública, 2012). On the other hand, WRAT-4 will be applied to evaluate math proficiency (Wilkinson and Robertson, 2006). For both reading and mathematical difficulties, IQ will be estimated through the WISC-IV battery (Wechsler, 2007). All psychometric evaluations are described in Table 4.

**TABLE 3:** Sample selection criteria.

Inclusion	
• Students from third to sixth grade • Evidence of low performance in reading or mathematics • To have daily access to an electronic device • Indistinct gender • Indistinct socioeconomic level	
**Exclusion**	**Suspension**
• Suffering from intellectual disability • Incapacity in reading or performing mathematics • Parents deny children participation	Withdrawal from the participant • Negative emotions from the child at any study stage

**TABLE 4:** Psychometric tests.

Session	Test name	Area to evaluate	Features to be assessed	How features will be assessed?	Estimated time
1	PREDISCAL (Pina et al., 2020)	Reading	Reading comprehension	Completing sentences	3 min
		Mathematics	Math fluency	Solving addition and subtraction	1 min
			Calculation	Solving equalities	3 min
	Words dictation (Zhang et al., 2020)	Reading	Spelling	30-words dictation	15 min
	d2-R (Brickenkamp et al., 2022)	Attention	Concentration	Selective attention task	15 min
2	WRAT-4 (Wilkinson and Robertson, 2006)	Mathematics	Arithmetic	40-exercises solution	15 min
			Fractions		
			Basic algebra		
	MPPERCC (Secretaría de Educación Pública, 2012)	Reading	Reading speed	Words read per minute	15 min
			Errors in reading	Words read erroneously and without spontaneous correction	
			Reading comprehension	Reading comprehension questions	
	WISC-IV (Wechsler, 2007)	IQ	Spatial relation	Designing different geometric patterns using red and white cubes	15 min
			Lexical knowledge	To formulate concepts and express the meaning of some words	

The tests will be applied in two sessions: (a) collectively and (b) individually, to evaluate reading and mathematical proficiency, as well as attentional levels. MPPERCC, manual of procedures for the promotion and evaluation of reading competence in the classroom; IQ, intellectual quotient.

Since the sample will be composed of children, parents or legal tutors must consent to the child participation. Participation of the children will be voluntary. Both parents or tutors and children must sign an informed consent, where the objectives of the study will be explained.

### 2.2 EEG system

EEG signals will be collected using the g.USBamp^3^ system (gtec, Austria) with a sample frequency of 256 Hz. A 32-channel setup will be used according to the 10/20 international system (Fp1, Fp2, F7, F3, Fz, F4, F8, FT9, FC5, FC1, FC2, FC6, FT10, T7, C3, Cz, C4, T8, CP5, CP1, CP2, CP6, TP9, P7, P3, Pz, P4, P8, TP10, O1, Oz, and O2). Left earlobe and Fpz channel will be set as reference and ground, respectively.

### 2.3 Experimental procedure

This study will be composed of four sessions. In the first two sessions, psychometric tests will be applied to identify low academic performance in reading or mathematics in students from third to sixth grade of elementary school. In session 1, children will be assessed collectively to estimate their reading and mathematical proficiency, as well as their attentional levels. These results will be used as a screening test to determine children capabilities in reading or mathematics. Then, in session 2, each child will be evaluated individually according to the learning difficulty identified in session 1. Subsequently, low-achieving children will be assigned to one of two categories:

• Category 1: Low academic performance in reading
• Category 2: Low academic performance in math

After that, the first EEG acquisition will be conducted in session 3. Children in category 1 will be randomly allocated in one of the following groups:

• Group 1: Children with low performance in reading who will receive reading training with Smartick (experimental group for reading)
• Group 2: Children with low performance in reading who will not receive any training with Smartick (control group for reading)

Similarly, category 2 will be randomly assigned as follows:

• Group 3: Children with low performance in math who will receive math training with Smartick (experimental group for math)
• Group 4: Children with low performance in math who will not receive any training with Smartick (control group for math)

**FIGURE 1:**
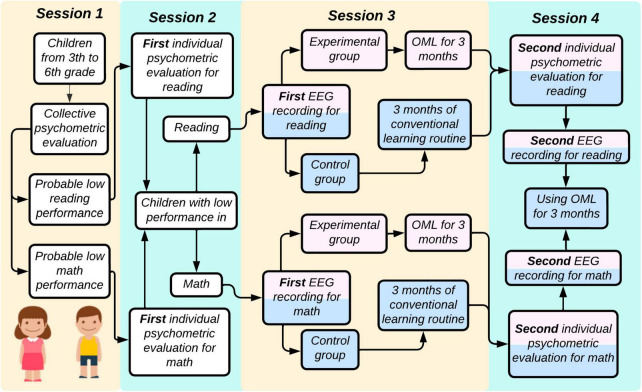
Experimental procedure. Four sessions will be executed throughout this study. Collective and individual psychometric evaluations will be carried out in session 1 and 2. As a result, children will be randomly assigned into one of the following groups: (a) experimental group for reading, (b) control group for reading, (c) experimental group for math, and (d) control group for math. In session 3, a first EEG will be obtained. After that, experimental groups will receive the learning training with Smartick for 3 months, whereas control groups will continue with their conventional learning routine for 3 months. In session 4, an individual psychometric evaluation and a second EEG recording must be performed after the 3 months. Color code: white square, no group assignment; pink square, experimental group; blue square, control group. EEG, electroencephalography; OML, online method for learning.

Children in category 1 and 2 will be executing reading and mathematical activities during EEG acquisition, respectively. Moreover, if the child volunteer is not willing to collaborate in the study, he/she will not be forced to carry out any test. Afterward, both experimental groups will start using Smartick after the first EEG session. Parents will be requested to ensure that the child undertakes a minimum of five sessions per week. Conversely, control groups will not receive any training and they will continue with their traditional learning method for 3 months. Then, each child must attend session 4, which will consist of a second individual psychometric evaluation (as in session 2), and a second EEG recording (as in session 3). Finally, the psychophysiological data gathered from experimental and control groups will be contrasted. The whole experimental procedure is depicted in Figure 1, and a detailed explanation of each session is described below.

It is noteworthy that providing a diagnosis of a specific learning disability is out of the scope of this work. However, psychometric results will be shared with parents or tutor, so that corrective actions can be taken as required.

#### 2.3.1 Session 1: Collective psychometric evaluation

The aim of this session will be to identify reading and mathematical difficulties, as well as attentional levels, in Mexican students from 3rd to 6th grade of elementary school. For this purpose, three psychometric tests will be applied collectively: (a) d2-R, for attention (Brickenkamp et al., 2022), (b) PREDISCAL, as a screening test for reading and mathematical skills (Pina et al., 2020), and (c) a word dictation for spelling proficiency (Zhang et al., 2020). At the end of this session, children will be categorized into one out of two categories: low academic performance in (a) reading or (b) mathematics. Those children with no learning difficulties will be excluded from the study. Conversely, if the child shows both learning difficulties, only the most affected area will be considered. If both learning difficulties are exhibited, only the learning area with the greatest academic lag will be considered. Prior to the execution of the tests, it is essential to create an atmosphere of immediate trust between students and the evaluator. The collective psychometric tests are described below:

##### 2.3.1.1 Attentional levels: d2-R

**FIGURE 2:**
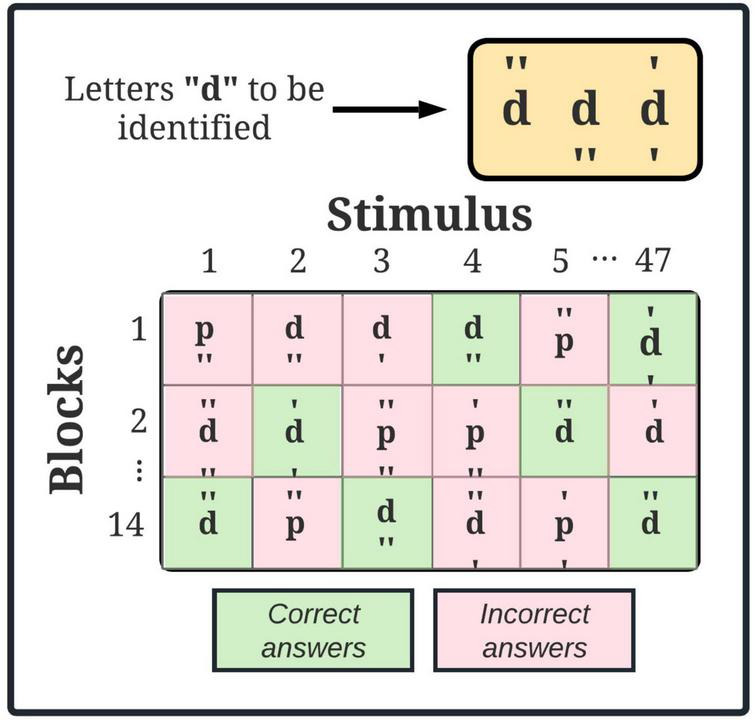
d2 test of attention. Yellow square, target stimulus; green square, target stimulus correctly identified; pink square, target stimulus incorrectly identified.

The d-2 test allows evaluating selective attention and concentration (Brickenkamp, 2009). The test comprises 14 blocks with 47 characters each, where letters “d” and “p” will be randomly presented. Each character will have from one to four markers around them. The target stimuli will be letter “d” with two marks as indicated in Figure 2. The duration for each block will be 20 seconds. Once the block is completed, it will no longer be possible to revert to the previous one.

##### 2.3.1.2 Reading and mathematical assessment: PREDISCAL

**FIGURE 3:**
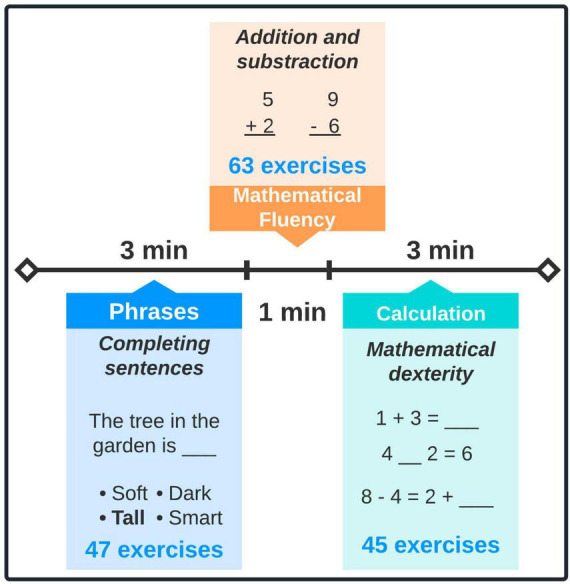
PREDISCAL test. This assessment encompasses three subtests, which evaluate reading and mathematical ability. PREDISCAL will last 7 min (plus instructions).

PREDISCAL is addressed to assess reading and arithmetical skills in children, aged between 7 and 12 years (Pina et al., 2020). PREDISCAL includes three subtests which assess: reading ability, mathematical fluency, and calculation. Regarding reading ability, decoding (grapheme-phoneme) and comprehension, syntax, basic orthography, and lexical knowledge are evaluated. On the other hand, elemental arithmetic operations (addition and subtraction) must be solved to assess mathematical fluency. Similarly, the calculation section attempts to examine mathematical dexterity by solving equivalences and equalities exercises. The PREDISCAL test is summarized in Figure 3.

##### 2.3.1.3 Spelling proficiency: dictation task

This test is addressed to estimate orthographic knowledge in children. It consists in the dictation of 30 words (see Table 5), which have been found to be confusing during elementary education (Zhang et al., 2020). Each word will be dictated one by one, without repeating them. When finished, the evaluation sheets will be collected.

**TABLE 5:** Dictation task.

#	Word	#	Word	#	Word
1	Circo	11	Esclavo	21	Huella
2	Cuervo	12	Cerveza	22	Bailarina
3	Casco	13	Dulce	23	Temblor
4	Volcán	14	Gitana	24	Hormiga
5	Cable	15	Lagarto	25	Castillo
6	Manzana	16	Uvas	26	Incendio
7	Parque	17	Cazador	27	Ángel
8	Pastel	18	Diente	28	Cine
9	Playa	19	Hueso	29	Brazo
10	Guitarra	20	Magia	30	Borrego

Spelling proficiency is to be evaluated with this assignment.

#### 2.3.2 Session 2: Individual psychometric evaluation

Those children who had low academic performance in the previous session will be individually evaluated. Additional reading and mathematical assessments will be applied to children with low academic performance in reading and mathematics, respectively. Once individual psychometric evaluations are finished, a sample of 112 children is expected to be recruited, which will be classified in four groups: (a) Group 1: 28 children with low performance in reading who will receive reading training with Smartick (experimental group for reading), (b) Group 2: 28 children with low performance in reading who will not receive any training with Smartick (control group for reading), (c) Group 3: 28 children with low performance in math who will receive math training with Smartick (experimental group for math) and (d) Group 4: 28 children with low performance in math who will not receive any training with Smartick (control group for math). Psychometric individual tests are described below:

##### 2.3.2.1 Reading test: manual of procedures for the promotion and assessment of reading competence in the classroom (MPPARCC)

Manual of procedures for the promotion and assessment of reading competence in the classroom (MPPARCC) is a protocol to assess reading speed, errors in reading, and reading comprehension in Mexican children. This tool is aimed at students from 1st grade of elementary school to 3rd grade of middle school (Secretaría de Educación Pública, 2012). This manual provides different texts to evaluate reading speed, where words read per minute will be considered. To implement this manual, a text will be presented to the child and then asked to read it aloud. The evaluator will measure the time it takes the child to read the whole text, and words per minute must be counted. Meanwhile, errors in reading must be identified (words modified during the reading and that were not corrected spontaneously). When the reading ends, reading comprehension will be assessed. The child will be asked three multiple-choice questions about the text. Finally, the child must tell what the story was about. The evaluation parameters for reading speed are mentioned in Table 6.

**TABLE 6:** Achievement levels for reading speed.

Scholar grade	Levels			
	Requires support	Comes close to standard	Standard	Advances
**Achievement levels for reading speed. Words read per minute.**				
3^rd^	< 60	≥ 60 and ≤ 84	≥ 85 and ≤ 99	> 99
4^th^	< 85	≥ 85 and ≤ 99	≥ 100 and ≤ 114	> 114
5^th^	< 100	≥ 100 and ≤ 114	≥ 115 and ≤ 124	> 124
6^th^	< 115	≥ 115 and ≤ 124	≥ 125 and ≤ 134	> 134

The ranges of words read per minute from 3rd to 6th grade are shown. Children in the “requires support” and “comes close to standard” levels will be considered for the study.

##### 2.3.2.2 Mathematical test: WRAT-4

The purpose of this test is to evaluate mathematical ability so that difficulties in calculation can be identified (Wilkinson and Robertson, 2006). WRAT-4 comprises 40 exercises including counting, simple arithmetic and fraction operations, and basic algebra. WRAT-4 sheets will be distributed among children, and they will have 15 min to answer as many exercises as possible. Children with average or below-average performance will be considered for this study.

##### 2.3.2.3 IQ evaluation: WISC-IV

The WISC-IV evaluation (Wechsler, 2007) will be used to estimate IQ through two subtests: vocabulary (VB), and block design (BD) (Sattler, 2010). Regarding VB, the child will be asked to name four different images and to define some words, whereas in BD different geometric patterns must be reproduced, by using red and white cubes.

#### 2.3.3 Session 3: Use and monitoring of the OML

**FIGURE 4:**
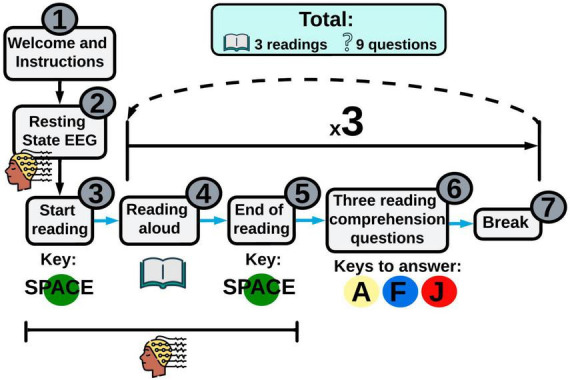
Experimental paradigm for reading. Reading evaluation will consist of displaying three different texts where the child must read carefully and aloud. The spacebar will have a green sticker, which must be pressed to start and end the reading. When the reading is finished, children must answer three reading comprehension questions with three possible answers each. Hence, children will choose one out of three options: (a) yellow (Key A), (b) blue (Key F) and (c) red (Key J).

**FIGURE 5:**
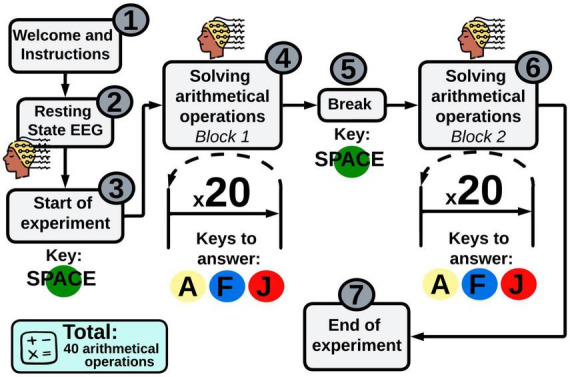
Experimental paradigm for mathematics. Mathematical assessment involves solving 40 arithmetical operations (addition and subtraction), divided into 2 blocks. Children must press A (yellow sticker), F (blue sticker) or J (red sticker) key to answer each operation.

Participants must attend a first EEG session, where reading or mathematical tasks will be performed simultaneously with the EEG recording. Therefore, two separate EEG experimental paradigms will be designed, including reading (see Figure 4) and mathematical (see Figure 5) activities. Both paradigms will be created by using Psychopy v2022.2.4 (Peirce et al., 2019) and OpenVibe 3.3.1 (Renard et al., 2010), which are software widely used in Psychology and Neuroscience for experiment design. Reading and mathematical paradigms are explained below.

##### 2.3.3.1 Experimental paradigm—Reading

Electroencephalography (EEG) signals will be collected while children are reading three different texts. Before starting, a resting state EEG recording will be taken for 3 min (Jäncke and Alahmadi, 2016; Rogala et al., 2020), while the child is staring at a “X” in the screen. After that, each reading will be shown. The child will be asked to read every text aloud, according to their reading abilities. After each reading, three multiple choice comprehension questions will be displayed, where children must select 1 out of the 3 options. A brief break will be given to the participants at the end of each reading. Every EEG recording from children who do not finish the reading will be discarded.

##### 2.3.3.2 Experimental paradigm—Mathematics

A 3-min resting state will be recorded at the beginning of the experiment. Then, children will perform 2 blocks of 20 arithmetical operations each. EEG signals will be recorded in the process. The participant will get a short break after completing the first block. As in the reading paradigm, children must answer every mathematical operation by selecting one out of three possible answers: yellow, blue, or red stickers. EEG recordings gathered from children who solve less than 50% correct per block will be discarded.

##### 2.3.3.3 Smartick

Smartick is an OML designed in Spain in 2009 by Arroyo and González de Vega (2009). Smartick has two gamified sections: (a) reading and (b) mathematics. Both platforms are aimed at improving reading and mathematical proficiency, respectively. Each interface is AI-based where the difficulty of the activities is adjusted according to each child capability: as user demonstrates proficiency, the system elevates the challenge level, whereas, in the presence of errors, it adeptly lowers the difficulty to optimize the learning experience.

All sessions of Smartick are customized according to the needs of each child. The better performance the child has, the higher score he/she receives. This score consists in star points called ticks, which can be exchanged for many virtual prizes within Smartick. It is noteworthy that Smartick allows parents, tutors, and researchers to supervise user activity (i.e., login day and time, number of right answers and ticks earned). However, it is imperative that parents or tutor do not assist the child in solving the activities.

This research is sponsored by the Smartick company, which will grant free access to the participants involved in this study. Smartick licenses will last for 3 months, where children must interact with the app for 15-min daily. In previous studies, it has been demonstrated that this time is enough to elicit positive effects in children rehabilitation (Wuang et al., 2018; Benzing and Schmidt, 2019).

Experimental groups will be using Smartick during the 3 months after the first EEG session. For these groups, (a) average score, (b) average usage time, and (c) total days of use will be considered as behavioral data to evaluate the performance of the children. Conversely, control groups will not use Smartick, however, they will continue with their traditional learning method for 3 months. When the 3-month period is over, Smartick will be given to these children, so that learning training can be performed.

##### 2.3.3.4 Session 4: Psychophysiological evaluation of the OML

Once the use of Smartick is finished, a second EEG and individual psychometric evaluations will be conducted. The second EEG will be taken, following the experimental paradigms described in session 3 (see Figures 4, 5). Similarly, the second psychometric evaluation is referred to session 2 (see Section “2.3.2 Session 2: Individual psychometric evaluation”). Psychophysiological changes are expected to be identified between children who will use Smartick and children who will not, regardless of the learning difficulty.

### 2.4 Study variables

Psychophysiological effects elicited in learning will be studied in children who will use and will not use an OML (independent variable). The experiment design will consider four study groups:

• Experimental groups, children with low academic performance in:

1. Reading
2. Mathematics

who will receive the learning training with Smartick.

• Control groups, children with low academic performance in:

3. Reading
4. Mathematics

who will not receive the learning training and will continue with their traditional method of learning.

Each child will be evaluated by the psychometric tests described in Table 7. These results (dependent variable 1) will determine which learning difficulty will be assisted by Smartick. From this technology, user performance (dependent variable 2) will be monitored to determine how the learning training improves the child’s proficiency. Moreover, EEG activity will be taken from both the experimental and control groups. This will allow the extraction of brain patterns (dependent variable 3) for the identification of neuroplastic changes associated with learning improvement. The study variables for each group are summarized below:

**TABLE 7:** Dependent variables for experimental and control groups.

Session	Evaluation		Variables	Description		
				**Type**	**Category**	**Rank**
1	PREDISCAL		Test score	Categorical	Severe	1–10
					Moderate	11–30
					No difficulty	31–70
					High performance	71–99
	Word dictation		Total of spelling errors	Numerical		
	D2-R		Test score	Categorical	Low	≤ 77
					Low-average	78–92
					Average	93–107
					High-average	108–122
					High	≥ 123
2	Reading	MPFVCL	Reading speed	Numerical		
			Errors in reading			
			Reading comprehension			
	Math	WRAT-4	Test score	Categorical	Extremely low	≤ 69
					Very low	70–79
					Low-average	80–89
					Average	90–109
	IQ	WISC-IV			High-average	110–119
					Very high	120–129
					Extremely high	≥ 130
3	Use of Smartick		User performance	Numerical	Average score	
					Average usage time	
					Total days of use	
	EEG		Brain pattern extraction	Numerical		
4	Word dictation		Total of spelling errors	Numerical		
	Reading	MPFVCL	Reading speed	Numerical		
			Errors in reading			
			Reading comprehension			
	Math	WRAT-4	Test score	Categorical	Extremely low	≤ 69
					Very low	70–79
					Low-average	80–89
					Average	90–109
					High-average	110–119
					Very high	120–129
					Extremely high	≥ 130
	EEG		Brain pattern extraction	Numerical		

The variables to be measured in children will consist of (a) psychometric test scores, (b) user performance within Smartick, and (c) a neurophysiological pattern extracted from EEG signals. EEG, electroencephalography; MPPERCC, manual of procedures for the promotion and evaluation of reading competence in the classroom; IQ, intellectual quotient.

### 2.5 EEG signal analysis

**FIGURE 6:**
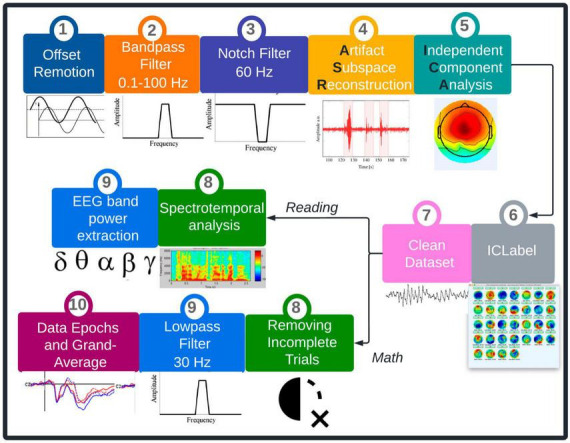
EEG signal analysis proposal. EEG data will come from children with low academic performance in reading or math. The preprocessing process (steps 1–7) will be the same for all groups. Conversely, both reading (steps 8–9) and math (steps 8–10) data will have independent signal processing.

Electroencephalography (EEG) signals from all children will be analyzed by the following three steps: (a) preprocessing, and (b) processing. Both processes are depicted in Figure 6.

#### 2.5.1 Preprocessing

Electroencephalography (EEG) data will be preprocessed in EEGLab toolbox (Delorme and Makeig, 2004) from MATLAB (The MathWorks Inc, 2020). An automated code will be created to clean EEG signals. First, DC offset will be removed, and the EEG data will be bandpass-filtered between 0.1 and 100 Hz. Additionally, a Notch filter for 60 Hz elimination will be implemented. After that, Artifact Subspace Reconstruction will be used to remove bad channels and exogenous artifacts. Finally, ICLabel will be used to eliminate muscular, cardiac, ocular, and line noise artifacts.

#### 2.5.2 Processing

Since reading and mathematics are two independent cognitive processes, EEG data will be processed separately. For the reading paradigm, spontaneous EEG activity will be analyzed, where only pure reading activity will be recorded. Thus, a spectro-temporal analysis is proposed in terms of absolute and relative power, which can reflect a relationship to differentiate brain activity among children who will use and will not use Smartick. Regarding the mathematics paradigm, ERP will be used to determine EEG patterns related to mathematical processing. ERP will be obtained at the beginning of each arithmetical operation, just in the moment when each operation appears on the screen. Also, the response time will be calculated for each operation.

### 2.6 Statistical analysis

Statistical significance (*p* < 0.05) in brain activity before and after Smartick will be identified. EEG data will be taken with the experimental paradigms described in Sections “2.3.3.1 Experimental paradigm—Reading” and “2.3.3.2 Experimental paradigm—Mathematics.” The first EEG acquisition will be performed in session 3, where four groups will be encountered:

• Group 1: Children with low performance in reading who will receive reading training with Smartick (experimental group for reading).
• Group 2: Children with low performance in reading who will not receive any training with Smartick (control group for reading).
• Group 3: Children with low performance in math who will receive math training with Smartick (experimental group for math).
• Group 4: Children with low performance in math who will not receive any training with Smartick (control group for math).

Changes in brain activity are expected in the experimental groups, which may be significantly different than the control groups. Therefore, statistical analysis will be carried out by pairs, as stated below:

• Pair 1: Group 1 and Group 2
• Pair 2: Group 3 and Group 4

**FIGURE 7:**
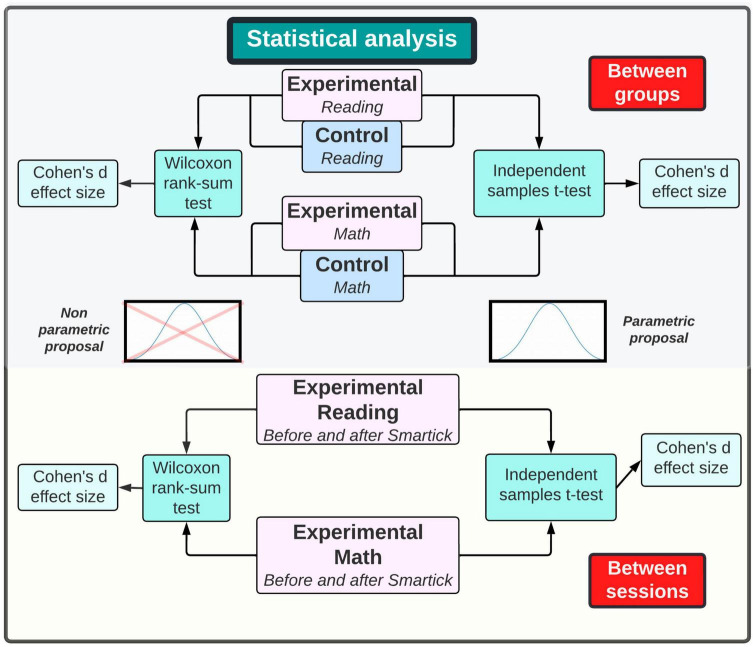
Statistical analysis proposal. Statistically significant differences will be determined among EEG recordings before and after Smartick.

After the first EEG recording, experimental and control group of each category will be contrasted. Firstly, normality is to be determined for both pairs. If normality is satisfied, an independent t-student for two samples test will be applied. Otherwise, a Wilcoxon Sum-Rank test will be utilized. Additionally, effect size will be calculated through EEG channels to verify the magnitude of the differences. When the second EEG is taken, experimental groups will be compared before and after using Smartick to determine if improvements in reading or mathematics were encountered. The statistical analysis proposal is shown in Figure 7.

## 3 Discussion

This research aims to evaluate an OML called Smartick, which includes training programs for the improvement of reading and mathematical skills. The technology will be assessed by psychophysiological data that encompasses (a) psychometric tests to valuate reading and mathematical proficiency, attentional levels, and IQ, (b) EEG data that will be collected when reading and mathematical activities are being performed, and (c) behavioral data, through the user performance within Smartick. This methodology will be undertaken in four sessions: (a) collective psychometric evaluation, where children will go through different psychometric tests in order to determine overall performance in reading and math, (b) individual psychometric evaluation, whose assessment will be specifically for the deficient area found in previous session, (c) use and monitoring of the OML, in which a first EEG recording will be taken before the implementation of the 3-month period of Smartick, and (d) psychophysiological evaluation of the OML, where a second EEG and psychometric evaluations will be carried out, so that indicative parameters of learning enhancement due to Smartick can be detected.

As previously stated, different studies have been performed to correlate EEG data with reading and mathematical learning difficulties. Several reports have shown increasing power in delta (Arns et al., 2011), theta (Arns et al., 2011; Soltanlou et al., 2019), alpha and beta bands (Che Wan Fadzal et al., 2012a,b). Similarly, an increment in power ratios from theta/alpha (Clarke et al., 2002; Jäncke and Alahmadi, 2016; Pavithran et al., 2019; Nassehi et al., 2020), and theta/beta (Clarke et al., 2002; Jäncke and Alahmadi, 2016) was obtained in children with learning difficulties. These results suggest that children with learning difficulties manifest higher rates of theta activity, which is associated with developmental delay (Pop-Jordanova, 2012), such as inattention, and poor reaction time and calculation (Demos, 2005). In addition, various analyses have been implemented to distinguish EEG neuromarkers related to reading and math learning, such as SVM (Frid and Manevitz, 2018; Perera et al., 2018; Mahmoodin et al., 2019), approximate entropy (Andreadis et al., 2009), ERP (Frid and Manevitz, 2018; Proverbio and Carminati, 2019), wavelet (Mahmoodin et al., 2019), and coherence (González-Garrido et al., 2018).

To improve cognitive skills in children with learning difficulties, a wide variety of digital applications have been designed and applied as AT. Prior studies have developed some applications to improve learning proficiency in (a) reading skills (Dyslexia Baca, DytectiveU, EasyLexia, Namagi, Alphabetics, AlphaTots), (b) mathematics (Talasia, Calculic Kids, MathFun, Coolmath4kids), or (c) both (Cognifit, Starfall). Furthermore, alternative research has suggested additional technologies such as VR (Hwang and Hu, 2013; Maskati et al., 2021; Rodríguez-Cano et al., 2021), AR (Ho et al., 2011; Vinumol et al., 2013; Avila-Pesantez et al., 2019; Gupta et al., 2019; Tenemaza et al., 2019; Kellems et al., 2020; Miundy et al., 2020), and eye-tracking (Katona, 2023) to enhance learning abilities.

So far, there are few neuroimaging studies about learning improvement due to AT (Kucian et al., 2011; Eroglu et al., 2022). The majority of studies solely conduct subjective assessments, such as surveys or questionnaires to describe user experience. Therefore, this protocol will allow monitoring Mexican children who will use Smartick, so that a follow-up psychophysiological evaluation can be performed to show if the OML caused learning enhancement. Previous research has demonstrated that using learning technology helps to increase motivation and improves self-efficacy and self-confidence (Kovari and Katona, 2023). This work could be applied in other Spanish-speaking countries that wish to evaluate AT for learning in children. However, non-Spanish-speaking countries must implement other types of psychometric evaluations that assess reading and calculation skills according to the native language.

## Ethics statement

The Ethics Committee from the Neuroscience Institute of University of Guadalajara approved this protocol on 28 April 2023, under this number: ET122022-356.

## Author contributions

CC-G: Conceptualization, Funding acquisition, Methodology, Project administration, Software, Writing – original draft. MR-F: Conceptualization, Methodology, Writing – review & editing. LA-V: Conceptualization, Funding acquisition, Methodology, Supervision, Writing – review & editing. DI-Z: Conceptualization, Methodology, Supervision, Writing – review & editing. VI-G: Conceptualization, Methodology, Writing – review & editing.

## Footnotes

1
https://mx.smartickmethod.com/

2
https://www.psychologie.hhu.de/arbeitsgruppen/allgemeine-psychologie-und-arbeitspsychologie/gpower

3
https://www.gtec.at/product/gusbamp-research/
